# A Novel Lineage of Proteobacteria Involved in Formation of Marine Fe-Oxidizing Microbial Mat Communities

**DOI:** 10.1371/journal.pone.0000667

**Published:** 2007-08-01

**Authors:** David Emerson, Jeremy A. Rentz, Timothy G. Lilburn, Richard E. Davis, Henry Aldrich, Clara Chan, Craig L. Moyer

**Affiliations:** 1 American Type Culture Collection, Manassas, Virginia, United States of America; 2 Department of Microbiology, University of Florida, Gainesville, Florida, United States of America; 3 Woods Hole Oceanographic Institute, Woods Hole, Massachusetts, United States of America; 4 Department of Biology, Western Washington University, Bellingham, Washington, United States of America; Portland State University, United States of America

## Abstract

**Background:**

For decades it has been recognized that neutrophilic Fe-oxidizing bacteria (FeOB) are associated with hydrothermal venting of Fe(II)-rich fluids associated with seamounts in the world's oceans. The evidence was based almost entirely on the mineralogical remains of the microbes, which themselves had neither been brought into culture or been assigned to a specific phylogenetic clade. We have used both cultivation and cultivation-independent techniques to study Fe-rich microbial mats associated with hydrothermal venting at Loihi Seamount, a submarine volcano.

**Methodology/Principle Findings:**

Using gradient enrichment techniques, two iron-oxidizing bacteria, strains PV-1 and JV-1, were isolated. Chemolithotrophic growth was observed under microaerobic conditions; Fe(II) and Fe^0^ were the only energy sources that supported growth. Both strains produced filamentous stalk-like structures composed of multiple nanometer sized fibrils of Fe-oxyhydroxide. These were consistent with mineralogical structures found in the iron mats. Phylogenetic analysis of the small subunit (SSU) rRNA gene demonstrated that strains PV-1 and JV-1 were identical and formed a monophyletic group deeply rooted within the *Proteobacteria*. The most similar sequence (85.3% similarity) from a cultivated isolate came from *Methylophaga marina*. Phylogenetic analysis of the RecA and GyrB protein sequences confirmed that these strains are distantly related to other members of the *Proteobacteria*. A cultivation-independent analysis of the SSU rRNA gene by terminal-restriction fragment (T-RF) profiling showed that this phylotype was most common in a variety of microbial mats collected at different times and locations at Loihi.

**Conclusions:**

On the basis of phylogenetic and physiological data, it is proposed that isolate PV-1^T^ ( = ATCC BAA-1019: JCM 14766) represents the type strain of a novel species in a new genus, *Mariprofundus ferrooxydans* gen. nov., sp. nov. Furthermore, the strain is the first cultured representative of a new candidatus class of the *Proteobacteria* that is widely distributed in deep-sea environments, Candidatus ζ *(zeta)*-*Proteobacteria* cl. nov.

## Introduction

Bacteria are known for the wide variety of reduced inorganic substrates they are able to oxidize and utilize as energy sources for growth. These include sulfur, ammonia, methane, hydrogen, and iron. The oxidation of ferrous (Fe (II)) to ferric (Fe(III)) iron is especially enigmatic because thermodynamically it yields a minimal amount of energy for growth, approximately ΔG°′ = −109 kJ mol^−1^, and at neutral pH chemical oxidation of Fe(II) to Fe(III), or iron oxyhydroxides, i.e. rust, occurs rapidly in the presence of oxygen [1]. To circumvent these problems, bacteria that oxidize Fe(II) to obtain energy at circumneutral pH have two key requirements for growth, low O_2_ partial pressures and sustained Fe(II) concentrations. This kind of metabolism has received scant attention in marine systems, first, because practically nothing is known about marine iron-oxidizing bacteria, and second, because the oceans are generally considered to be oxygenated and depleted in Fe(II). In addition, marine sediments tend to be rich in sulfide, which reacts rapidly with Fe(II) to precipitate FeS.

An important exception to these oceanic iron depleted conditions occurs in areas of hydrothermal venting, either at seamounts or at crustal spreading centers. Here, anoxic vent fluids charged with Fe(II) come in contact with the cold, oxygenated ocean water. It is estimated that the present-day flux of Fe(II) from hydrothermal venting is approximately 3×10^11^ mol yr^−1^ [2]. As a result, there are often substantial deposits of Fe-oxides at these vents, and in nearly every case that has been examined, the morphology of Fe-oxides provide abundant evidence for microbial activity [3], [4], [5], [6], [7]. The tell-tale signs of this activity are the tubular sheath casts of filamentous Fe-oxidizing bacteria (FeOB), or the twisted stalks of *Gallionella*-like organisms. Another potential habitat for marine FeOB is freshly formed oceanic basalts that result from volcanic and/or tectonic activity. In this case, leaching of Fe(II) directly from the mineral or glass surfaces may occur allowing for growth of biofilms of FeOB on the weathered rocks [8], [9].

These types of habitats are more common than is often appreciated, since crustal spreading centers and subduction zones, as well as seamounts with either active volcanism or cold seepage of mineral rich fluid flow are common throughout the world's oceans [10]. Because iron is the fourth most abundant element in the Earth's crust, it is often the most abundant potential energy source at the boundaries of the oxygenated ocean and the reducing subsurface [11]. However, we know very little about the population biology of prokaryotic lithotrophic communities that could potentially live off the Fe(II)-rich waters and vent fluids associated with these kinds of boundary habitats [1], [12].

We previously reported on the role that lithotrophic FeOB appear to play in the formation of extensive microbial mats that form at the Loihi Seamount [13]. During that study two novel FeOB were isolated, strains JV-1 and PV-1. These organisms were striking because as they grew, they formed filamentous stalk-like structures that appeared to consist primarily of an amorphous iron oxyhydroxide. These filaments bore a strong morphological resemblance to the types of oxides seen in the mats at Loihi[13]. We report here a more complete characterization of these two isolates and show that their metabolism is limited to Fe-oxidation. A cultivation-independent approach confirmed that they are common in iron mats located at Loihi. They are phylogenetically novel and represent the first cultured isolates of a new class of *Proteobacteria* that until now has only been represented by sequences in environmental SSU rRNA gene libraries associated with the floor of the deep ocean.

## Results

### Growth characteristics of PV-1 and JV-1

Both strains grew microaerobically with Fe^2+^ as the sole energy source and CO_2_ as the only available carbon source indicating the strains were obligate, lithotrophic FeOB. The following inorganic and organic substrates did not support growth: ammonium, thiosulfate, sulfide, Mn(II), tetrathionate, hydrogen, formate, acetate, pyruvate, succinate, glucose, galactose, ribose, glycerol, aspartate, and glycine. In addition no growth either under aerobic or microaerobic conditions was observed for the following complex media: tryptic soy agar, marine agar, and R2A (Difco) supplemented with 2.5% NaCl, nutrient agar supplemented with 2.5% NaCl, blood agar, PYG medium supplemented with 2.5% salt, or thioglycollate medium also supplemented with NaCl. The strains had an absolute requirement for marine salts and did not grow in a freshwater Fe-oxidizing medium that contained <0.2 g/l Mg or Ca-salts. Both strains are motile. They prefer low oxygen concentrations and, based on their behavior in gradient tubes, appear to be aerotactic. Neither strain was able to reduce Fe(III) with acetate anaerobically, nor were they able to oxidize Fe(II) with nitrate as the electron acceptor. The isolates grew at temperatures between 10° and 30°C. Growth appeared to be very slow at 5°C and was not observed at 35°C. The pH range for growth was 5.5–7.2 and appeared optimal between 6.2 and 6.5. A spot test for catalase activity was negative.

**Figure 1:**
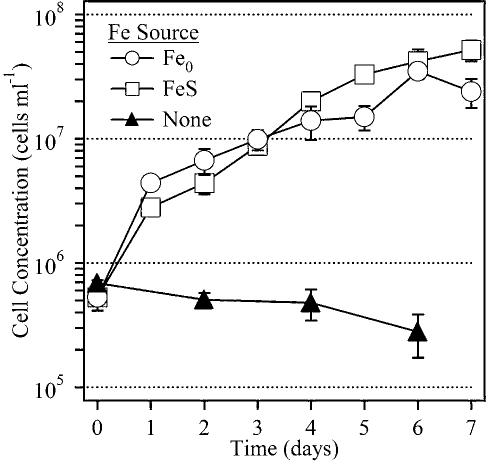
Growth curve of PV-1 on gradient plates with FeS (□) or Fe^0^ (○) as the iron source. A control (σ) with agarose but no FeS was included. Cell numbers were determined by direct counts and errors are based on duplicate samples.

These FeOB were able to grow on a variety of forms of Fe(II) including FeS or on FeCO_3_ (siderite) in gradient culture and on FeCl_2_, Fe(NH_4_)_2_(SO_4_)_2_, and FeSO_4_ in liquid bottle cultures. They were also able to grow on zero valent iron, Fe(0). A growth curve for strain PV-1 comparing growth on Fe(0) and FeS is shown in Figure 1. Final growth yields tended to be higher on FeS, but initial growth yields on both forms of iron were comparable. The doubling time at 23°C was on the order of 12 h.

### FAME analysis

The MIDI system was only able to identify approximately 45% of the fatty acids in lipid extracts from late-log phase PV-1 cells. The dominant fatty acid was 11:0 iso 3OH, followed in relative abundance by 17∶0 iso 3OH, 16∶0, and 18∶1 iso H (Table 1). Another dominant set of fatty acids was 16∶1 w7c/15 iso 2OH, however these two fatty acids could not be discriminated from one another and are reported as summed in Feature 3 in Table 1. The abundance of the branched chain fatty acid 11∶0 iso 3OH in PV-1 is unusual. This is generally considered a rare fatty acid in bacteria [14]. It has been shown to be diagnostic of the family *Xanthomonadaceae* and normally comprises <10% of the total fatty acids in these organisms [15], [16].

**Table 1:** FAME Analysis, showing fatty acids ≥2% of total.

Fatty Acid	Percent (%)		Average	Standard Error
	Run 1	Run 2		
12∶0 iso	2	2	2	0
11∶0 iso 3OH	27	28	27.5	0.5
15∶1 w8c	1	3	2	1
16∶0	14	9	11.5	2.5
18∶1 iso H	5	6	5.5	0.5
17∶0 iso 3OH	13	14	13.5	0.5
Summed in Feature 3^1^	26	23	24.5	1.5
Summed in Feature7^2^	6	7	6.5	0.5

1 16∶1 w7c/15 iso 2OH

2 19∶0 CYCLO w10c/19w6

### Morphology

**Figure 2:**
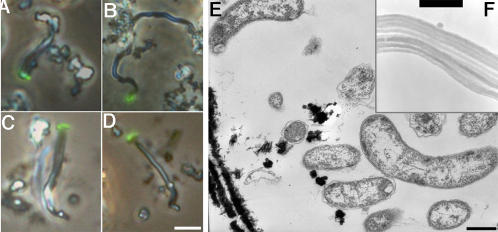
Micrographs of strain PV-1. A–D, Light micrographs showing cells attached at the end of the filaments; the cells have been stained with Syto and composite epifluoresence and phase contrast images produced to show the cells stained green. The bar = 5 µm. E TEM image of the cells, note the Gram-negative type cell wall and the fibers of Fe-oxides in the lower left corner. The bar = 0.5 µm. F. A HRTEM image of the Fe-oxide filament showing its composition as a bundle of conjoined fibers of Fe-oxide. The bar = 0.63 µm.

Cells of strains PV-1 and JV-1 were curved rods (approximately 0.5×2–5 µm). The most distinguishing characteristic of these strains was the filamentous stalk-like structures of iron oxyhydroxides that they formed during cell growth, Figure 2 and [13]. The cells had Gram negative type cell walls when viewed by TEM, Figure 2e, and the fibrillar Fe-oxyhydroxides were observed as well as lesser amounts of fine particulate oxides sometimes associated with the cells. HRTEM showed that the stalks were themselves composed of a set of discrete fibers aligned parallel to the length of the stalk, Figure 2f. The stalk shown in Figure 2f is between 0.6 and 0.7 microns wide, with individual fibers that are each ∼70 nm in diameter. The sample was not stained; thus contrast is due to iron minerals on or within the stalk. It was confirmed by energy dispersive X-ray analysis that the major element in these structures was Fe (data not shown). Treatment of the stalks with 0.3 M oxalic acid caused a rapid and nearly complete dissolution, suggesting that they are composed primarily of poorly crystalline iron oxyhydroxides (Supplemental Figure S1). When glass surfaces (microscope slides) were placed in a culture of PV-1 growing in liquid culture, the cells attached to the glass surface and formed stalks, Figure 2a–d. This confirmed that the cells grew at the apical ends of the stalks; presumably these cells are extruding the Fe oxyhydroxide filaments as they grow.

### Phylogeny

**Figure 3:**
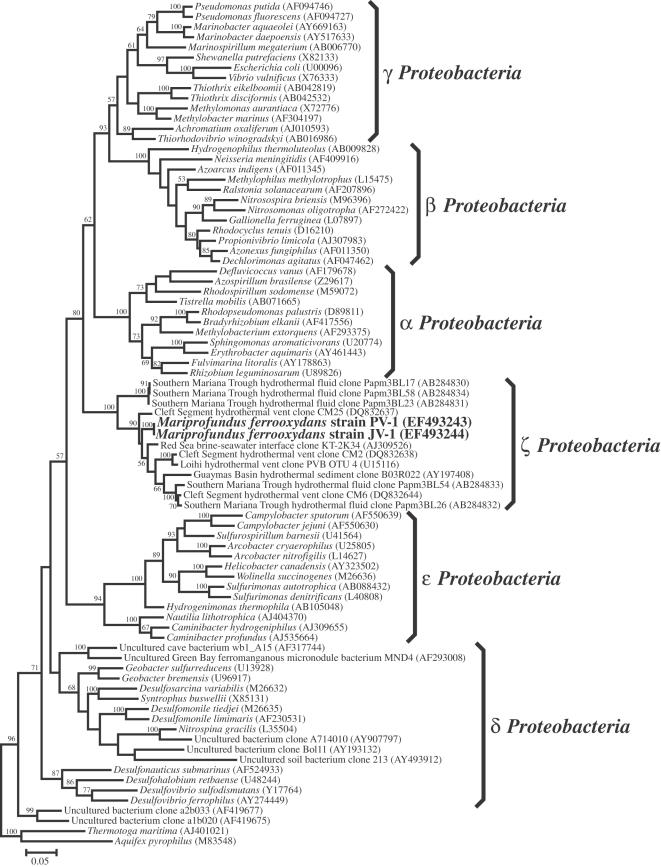
Maximum-likelihood phylogenetic tree showing the evolutionary placement of *Mariprofundus ferrooxydans* (strains PV-1 and JV-1) belonging to the novel class of zeta-Proteobacteria along with representatives from other previously described classes of Proteobacteria. Scale bar represents 5 nucleotide substitutions per 100 positions.

**Figure 4:**
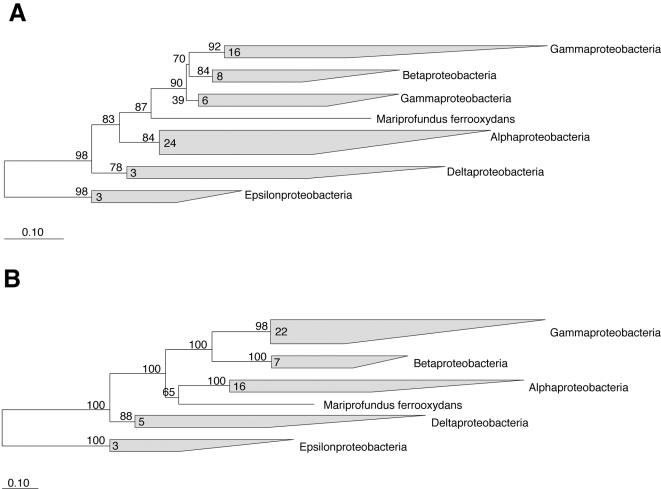
Two maximum likelihood trees showing the phylogenetic relationship of *Mariprofundus ferrooxydans* PV-1 to other *Proteobacteria* based on conserved protein sequences. Figures A and B show trees inferred from the amino acid sequences of the widely conserved RecA and GyrB proteins, respectively. The shaded clusters represent the recognized classes of *Proteobacteria*. The number of sequences representing each class is shown inside the cluster. The numbers at the nodes represent the per cent support for that group. The scale bar represents the estimated number of substitutions along the branches.

PV-1 and JV-1 SSU rDNA sequences were identical (1488 bases). Their genotypic similarity was confirmed with rep-PCR, where both strains gave nearly identical banding patterns (Supplemental Figure S2), that were distinct from freshwater FeOB. A phylogenetic analysis based on comparison of the SSU rRNA gene with other bacteria revealed that PV-1 and JV-1 were unusual members of the phylum *Proteobacteria* in that they did not cluster with any of the known classes of α-, β-, γ-, ε-, or δ-*Proteobacteria* (Figure 3) [17], [18]. Instead, the strains branched deeply within the *Proteobacteria* as shown in a phylogenetic tree created by maximum likelihood [19], Figure 3. Very similar tree topologies were found by neighbor-joining, maximum parsimony or minimum evolution methods using MEGA3 [20](data not shown). The novelty of the strain PV-1 within the *Proteobacteria* was confirmed by phylogenetic analysis of two other highly conserved proteins, gyrase beta-subunit (GyrB), and the recombination protein A (RecA). Phylogenetic trees for the GyrB and RecA proteins translated from the gene sequences are shown in Figure 4a and 4b, respectively. In both cases, PV-1 clustered within the *Proteobacteria*, but formed a distinct lineage from the other described classes of *Proteobacteria*.

**Figure 5:**
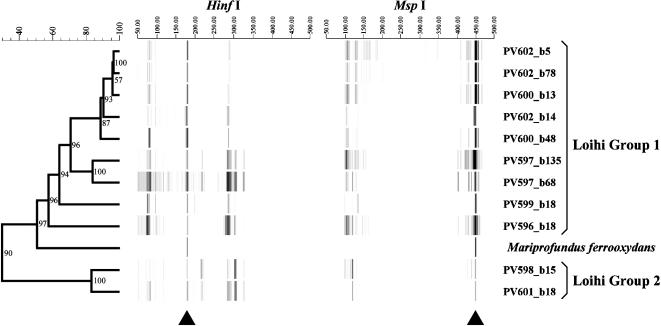
UPGMA/Pearson product-moment cluster analysis of T-RFLP bacterial community fingerprints using eight restriction digest treatments from iron-dominated mats from Loihi Seamount including the isolate *Mariprofundus ferrooxydans.* Two groups of communities have been identified and designated Loihi Group 1 (from lower temperature vents with enhanced ferrous iron) and Loihi Group 2 (from higher temperature vents with enhanced sulphide; ([37]). Triangles indicate T-RFs associated with *Mariprofundus ferrooxydans* from restriction digests with Hinf I and Msp I (176 bp and 449 bp, respectively). T-RF scales range from 50 to 500 base pairs. Numbers at the nodes indicate the cophenetic correlation value of the cluster. Dendrogram scale bar units are r value ×100.

A BLAST search of GenBank using the SSU rRNA gene sequence from PV-1 revealed a group of environmental clones that clustered within the PV-1/JV-1 lineage, Figure 3. All these clones were from deep-sea marine habitats, primarily associated with hydrothermal activity. A T-RFLP (terminal restriction fragment length polymorphism) analysis of Fe-rich microbial mats collected at several different vent sites around the summit of Loihi in 2004 found that the PV-1 phylotype was consistently present at all the sampled sites, Figure 5. The vent water temperatures at these sites ranged from 10° to 65°C, and there were two main T-RF clusters, Loihi Group I that was associated with cooler vents <40°C and Loihi Group 2 that was associated with vents >40°C. The PV-1/JV-1 phylotype appeared most strongly associated with the Group I populations, Figure 5. Light microscopy indicated the presence of filamentous Fe oxyhydroxides similar to those formed by PV-1 at all these sites as well (data not shown).

## Discussion

A systematic analysis of these two microbes from microbial iron mats at Loihi Seamount indicates that they appear to be the first cultured isolates of a candidatus class of *Proteobacteria*, the “ζ-*Proteobacteria*”. The most conspicuous trait of these two strains is their obligate dependence upon Fe(II) as an energy source and microaerobic conditions. They are incapable of heterotrophic growth; neither can they grow on other inorganic energy sources such as reduced S-compounds or H_2_. These strains are also obligate aerobes, they do not oxidize Fe(II) when grown anaerobically with nitrate, nor can they reduce Fe(III) in the presence of acetate. The ability of these strains to grow on Fe^0^ metal is of interest, as we are not aware of other reports of Fe-oxidizing bacteria growing on metallic iron, although there is evidence that there can be direct electron flow from Fe^0^ to support the growth of methanogens and obligately anaerobic sulfate-reducing bacteria [21]. Under aerobic conditions the reaction of iron metal is 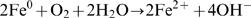
(1)Presumably the actual substrate for the FeOB is Fe(II) that is released upon chemical oxidation of the Fe(0); this process would be speeded up due to the ionic strength of seawater. Because these bacteria are growing under suboxic gradient conditions, O_2_ may not always be present, in which case the oxidant of of Fe^0^ may be water which results in hydrogen production: 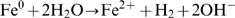
(2)However, since these bacteria have been shown not to use H_2_ as an energy source, their growth substrate will still be Fe(II). We have not yet demonstrated CO_2_ fixation by either PV-1 or JV-1, however both grow well without any added organic C source. The draft genome sequence of PV-1 (NZ_AATS00000000) reveals it has a gene for the small subunit ribulose-1,5-bisphosphate carboxylase/oxygenase (Rubisco) protein, as well as genes for both form I and form II of the large subunit Rubisco, indicating that PV-1 has the genetic complement necessary for autotrophic growth.

As was noted earlier, these marine Fe-oxidizers produce a unique filamentous stalk-like structure composed primarily of Fe-oxyhydroxides [13]. More detailed electron micrographic analysis presented here indicates that the Fe-containing stalks are themselves composed of bundles of iron-rich fibrils. The processes of how these fibrils are excreted and their subcellular localization are currently under investigation. While it is presumed that an organic matrix is excreted by the cells that may serve as a ‘scaffold’ for the formation of Fe-oxides, the composition of the matrix is unknown. Presumably it contains acidic polysaccharides that play a role in metal binding and coordination [22], [23]. At a gross morphological level there appears to be a remarkable congruence between the filamentous Fe oxyhydroxides produced by strains PV-1 and JV-1 and the ferrihydrite-rich stalks produced by *Gallionella ferruginea* [24]. *G. ferruginea* cells share a similar morphology to PV-1 and JV-1 cells, since they are also curved rods. The ferrihydrite-containing stalk of *G. ferruginea* is noted for its regular helical structure. Both PV-1 and JV-1 can produce twisted stalks, and at times these can appear helical; however as viewed by light microscopy, and shown in Fig 2 (a–d) they do not appear to have the regular helicity that is often observed with *G. ferruginea*. The overall morphological similarity between *G. ferruginea* and the PV-1 and JV-1 strains suggests that reports of *Gallionella spp.* from deep-sea marine habitats may need to be re-evaluated.

It is also interesting to note that certain lithoautotrophic S-oxidizing bacteria that have been found at hydrothermal vents also may produce copious filamentous sulfur structures [25]. The morphology of these sulfur filaments bears a notable resemblance to the iron stalks formed by strains PV-1 and JV-1; however, the organism identified with filamentous sulfur formation is an epsilon ε-*Proteobacteria, Candidatus* Arcobacter sulfidicus [26], and, as far as is known, does not oxidize iron. It will be interesting to determine if the mechanisms for deposition of iron-rich filaments and sulfur-rich filaments share any commonalities.

### Comparison to other neutrophilic FeOB

Edwards *et al.* [27] described two groups of marine FeOB that were most closely related to *Hyphomonas jannaschiana* and *Marinobacter aquaeolei*, which are α- and γ-*Proteobacteria*, respectively. These organisms can both grow heterotrophically using organic substrates, as well as lithoautotrophically, on Fe(II), although their doubling times are appreciably slower than either PV-1 or JV-1 on Fe(II). Neither of these organism forms a filamentous iron oxyhydroxide. Freshwater FeOB isolates described to date include *G. ferruginea*, as discussed above [28], FeOB TW2 [29], and two strains isolated from groundwater, ES-1, and ES-2 [30]; The latter two organisms have recently been described as ‘*Sideroxydans lithotrophicus*’ and ‘*Gallionella ferricapsiformans*’, novel members of the *β-Proteobacteria* [31]. Another group of obligately lithotrophic FeOB has been isolated from the rhizosphere of wetland plants. These organisms are also members of the *β-Proteobacteria* and include a new species, ‘*Sideroxydans paludicola*’ and a novel genus ‘*Ferrotrophicum radicicola*’ [32]. Like other freshwater isolates they do not grow with elevated salt concentrations, nor do they form morphologically unique Fe-oxides. Another differentiating feature of these marine FeOB is that the dominant fatty acid that was identified was the branched chain hydroxy 11:0 iso 3OH; this fatty acid was not found in the freshwater strains, instead the saturated straight chain fatty acids 14:0 and 16:0 were more prevalent in these isolates.

### Environmental Relevance

Several clones from deep-sea environmental samples were the closest relatives to PV-1 based upon SSU rRNA gene sequences retrieved through a BLASTN search (Figure 3). For near full-length sequences (>1200 bases), a clone from the Kebrit Deep (depth 1468m), in the Red Sea [33] was 95.4% similar (1373 bases) to PV-1 and a Loihi Seamount hydrothermal vent clone, Loihi Seamount PV*B* OTU4 [34], [35] was 93.4% similar (1455 bases). In addition to the Loihi clone, other related SSU rRNA gene signatures have been observed from deep-sea hydrothermal systems, including the Guaymas Basin [36], the Cleft Segment hydrothermal system off the coast of Oregon [37], and the Mariana Trench in the western Pacific. With the exception of the Moyer *et al*. [35]study that predated the current work at Loihi, none of the reports yielding related sequences were specifically investigating FeOB. However, the observation of sequences related to PV-1 from numerous hydrothermal systems suggests FeOB may be more widespread than is currently recognized [38], [39], [40].

Observation of iron-oxide filaments similar to those made by PV-1 in deep-sea sediments have also been made at the Axial Volcano of the Juan de Fuca Ridge [41] and Vailul'u Volcano near Samoa [7]. Representatives of the *Mariprofundus* group have also been detected by T-RFLP and clone library analyses of microbial mats from N.W. Eifuku Volcano along the Mariana Island Arc, as well as the Cleft Segment of the Juan de Fuca Ridge and have been shown to co-occur with characteristic Fe-rich filaments [37]. Numerous samples of microbial mats collected at different times from different vent sites from Loihi also share predominant populations that correspond with those expected from the PV-1/JV-1 phylotype (data not shown). These findings corroborate the T-RFLP analysis presented here (Figure 5), that show the *Mariprofundus* phylotype exists at numerous vent sites at Loihi.

### Description of *Mariprofundus* gen. nov

*Mariprofundus* gen. nov. (Mar.is.pro.fund'us. L. masc. n. *maris* the sea; L. adj. *profundus* deep; L. masc. n. *Mariprofundus* a deep-sea organism).

Cells are motile curved rods that appear Gram-negative by transmission electron microscopy. Growth is obligately lithotrophic and requires Fe^2+^ as the energy source. Growth is oxygen dependent and requires marine salts.

### Description of Mariprofundus ferrooxydans sp. nov

*Mariprofundus ferrooxydans* (ferr.ox'y.dans. L. n. *ferrum* iron; Gr. adj. *oxys* sour; N.L. v. *oxydo* to sour, oxidize; N.L. part. adj. *ferrooxydans* iron-oxidizing).

Displays the following properties in addition to those given by the genus description. Growth requires microaerobic conditions. The cells do not grow on other reduced inorganic energy sources besides iron or on organic compounds. As a result of growth filamentous stalk-like structures containing poorly crystalline iron oxyhydroxide are produced. The optimum growth temperature is 30°C and the optimum pH range is 6.0–6.5. The G+C content of the DNA of PV-1 is 54%. The cells are catalase negative. The dominant fatty acids are 11∶0 iso 3OH, 17∶0 iso 3OH, 16∶0, and 18∶1 iso H The type strain is PV-1^T^, ATCC™ BAA-1019 ( = JCM 14766) isolated from iron-rich microbial mats associated with regions of hydrothermal venting at Loihi Seamount in the Pacific Ocean. The class, order, and family description for this species are provided in the supplemental information (Supplemental Text S1).

## Materials and Methods

### Source and growth conditions

Both isolates were enriched from Fe-rich mats associated with hydrothermal venting at Loihi Seamount as described previously [13]. PV-1 came from an Fe-mat collected in 1996 associated with a cool (23°C), diffuse vent site, Naha Vents, located below the summit of the Loihi at a depth of 1325 m. JV-1 was isolated from an Fe-mat collected in 1998 that came from the lower jet at the Ikaika Vents (1298 m), a hot (165°C) vent located in a 300 m deep caldera close to the summit of Loihi. PV-1 was isolated directly from an inoculum of undiluted mat material, while JV-1 was enriched from a mat sample initially diluted to 10^–7^. Details of the isolation of JV-1 have been published previously and much the same techniques were used for the isolation of PV-1 [13].

The basal medium for all studies described here was artificial sea water (ASW) amended with nitrogen and phosphate sources according to the following formulation (per L deionized-H_2_O): 27.5 g NaCl, 6.78 g MgCl_2_ 7H_2_O, 5.38 g MgCl_2 _· 6H_2_O, 1.4 g CaCl_2_ · 2H_2_O, 1.0 g NH_4_Cl, 0.72 g KCl, 0.2 g NaHCO_3_, 0.05 g K_2_HPO_4_ · 3H_2_O, 1.0 ml trace mineral supplement, and 1.0 ml vitamin supplement (both from American Type Culture Collection). Sterile aliquots of the vitamins and mineral solutions were added after the mineral salts medium was autoclaved at 121°C for 20 min. Filter sterilized sodium bicarbonate (10 mM final concentration) was added to provide buffering capacity and a carbon source.

Growth of the organisms was done under conditions of limited, but constant, supply of Fe(II) and O_2_ using gradient tubes or plates, or bottle cultures. Details of these techniques have been published elsewhere [42]. They all share in common the provision of Fe(II) concentrations in the 50–300 µM range and O_2_ concentrations below 15% of ambient. One modification of the gradient plate technique was developed to test the ability of these strains to grow on zero valence iron (Fe^0^). This utilized autoclaved Fe^0^ particles as an iron source instead of the agarose stabilized FeS layer. Approximately 100 mg of Fe^0^ was added to 15 ml of ASW medium in a standard Petri plate, and the plate was gently swirled to distribute the Fe particles as uniformly as possible. The bacteria were inoculated into the liquid and the plates were incubated in Gaspak jars as previously described [13].

### Growth and phenotypic studies

Tests for growth on individual C-sources for both strains were done as described before for JV-1 [13]. Growth rates were determined in gradient plates. Twelve plates were inoculated and incubated in Gaspak jars at room temperature. Each day two plates were removed and the liquid medium (15 ml) from each plate was harvested into a 50 ml conical tube using a 10 ml pipet. The Fe-oxides were vortexed for 30 seconds and aliquots were removed for direct counts as described previously [13].

The pH range for growth of each strain was tested by varying the buffers used in the top layer of the gradient tubes. Sodium bicarbonate (10 mM, gassed with CO_2_) was used to buffer at pH 5.0, 5.5, and 6.5; 5 mM N-Cyclohexyl-2-aminoethanesulfonic acid (HEPES), adjusted with NaOH, was used at pH 7.0 and 7.5. Gradient tubes were considered positive or negative for growth after one week by the formation of a tight band of growth at the oxic-anoxic interface; growth was confirmed as presence of cells after staining with Syto 13 (Molecular Probes) and viewing by epifluoresence microscopy.

### Electron Microscopy

For standard transmission electron microscopy (TEM), PV-1 cells were grown on gradient plates with FeS to the late log phase of growth. The cells were harvested by centrifugation and resuspended in 20 mM HEPES buffer (pH 7.0) and fixed with 2.5% glutaraldehyde. The cells were then washed in 0.1 M sodium cacodylate buffer and fixed with 1% osmium tetroxide in the cacodylate buffer for 45 min on ice. Next the cells were washed with distilled water and stained with 1% aqueous uranyl acetate, then dehydrated in an ethanol series and embedded in Spurr resin. Thin sections were cut on an LKB Nova ultramicrotome, post-stained with uranyl acetate and lead citrate, and observed and photographed in a Zeiss EM-10CA TEM [43].

For high resolution transmission electron microscopy (HRTEM), a late log phase culture of PV-1 was grown in gradient plates and preserved with 2% gluteraldehyde. A few microliters of preserved sample was deposited onto a formvar-coated copper grid, rinsed with deionized water, and air-dried. The sample was coated with carbon and examined on a Philips CM200 transmission electron microscope, operated at an accelerating voltage of 200 kV.

### Fatty acids analysis

Fatty acid methyl esters (FAME) were identified using the procedure recommended by Microbial Identification System (MIDI, Sherlock Microbial Identification System Version 4.0, MIS Operating Manual, March 2001, Newark, DE). The cells were grown in gradient plates (100 ml total) until the late log phase of growth and then harvested by centrifugation. Excess Fe(III) was removed from the cell pellet by treating the sample with 0.33 M oxalic acid for one hour at 37°C, and then washing it three times by centrifugation with de-ionized water. The extraction of FAMEs and their analysis by gas chromatography was done as previously described [44].

### Phylogenetic Analysis

DNA was extracted from phosphate buffered (50 mM) saline (pH 8.0) washed pellets containing cells and iron oxides using the MoBio PowerSoil DNA Isolation kit. This minimal procedure produced DNA of high quantity and quality, which was not matched in previous studies [13], [30] due to low cell counts (10^7^ ml^−1^) and binding of DNA to iron oxide surfaces.

Universal primers 27F and 1492R were used to amplify the nearly full length SSU rRNA gene from the DNA extract. The PCR product was run on a 1% pre-cast SeaKem Gold Agarose gel (1× TBE buffer plus ethidium bromide from Cambrex Bio Science, ME). The specific band was excised from the gel and purified using a QIAquick Gel extraction kit (Qiagen Sciences, MD). The purified DNA was sequenced with a CEQ 8000 genetic analyzer (Beckman Coulter, CA).

For phylogenetic analysis, representative sequences were imported into the ARB software environment and aligned to the ARB database using the ARB fast aligner [45]. Additional sequences were selected in the database and exported to the sequence alignment program Bioedit [46] for additional alignment editing. Phylogenetic analyses were restricted to regions of moderately to highly conserved nucleotide positions that were unambiguously aligned for all sequences. Phylogenetic placements were calculated using fastDNAml version 1.2.2 [19] using the general two-parameter model of evolution [47] and allowing for the global swapping of branches. Using these parameters, the search for an optimal tree was repeated until the best log likelihood tree was calculated in at least three independent tree calculations. Each phylogenetic tree was bootstrapped 100 times allowing for the global swapping of branches. The search for each bootstrap was repeated until the best log likelihood score was calculated for at least two independent bootstrap calculations.

Phylogenetic analysis of the DNA topoisomerase gyrase subunit B (GyrB) protein, and the bacterial recombination (RecA) protein was carried out by extracting the appropriate DNAsequences from the draft genome sequence of PV-1. The genome (NZ_AATS00000000) was sequenced by the J Craig Venter Institute (www.VenterInstitute.org); details will be published elsewhere. The GyrB sequence was aligned with 53 homologous sequences from species in the *Proteobacteria*, using the L-INS-i option of the MAFFT program [48], [49]. A tree was inferred from 725 homologous positions using the TREEFINDER algorithm (www.treefinder.de). The Dayhoff model of amino acid substitution was used and the discrete gamma distribution model, with four rate categories and an estimated alpha parameter, was implemented. Branch support was estimated using the LRSH approximation [50] in TREEFINDER. The RecA sequence was aligned to 60 homologous sequences from the *Proteobacteria* and 317 positions were used to build the tree. The same software and parameters were used for aligning and inferring the tree. A list of the taxa used to construct both RecA and GyrB trees is provided in Supplementary Table S1.

Repetitive element PCR (Rep-PCR) was carried out using the Diversilab system from Bacterial Barcodes (www.bacbarcodes.com). PV-1 and JV-1 cells were grown in gradient plates, harvested, and subjected to DNA extraction as described above. The DNA was amplified using the *Stenotrophomonas* kit provided by Bacterial Barcodes, which contains a proprietary set of primers for repetitive elements common among *Proteobacteria*. The PCR products were separated on an Agilent 2100 Bioanalyzer, and a dendrogram comparing the new profiles to profiles from a database of samples was generated using the DiversiLab software from Bacterial Barcodes.

### T-RFLP analysis

Microbial mat samples were collected in 2004 using a suction sampling apparatus (Emerson and Moyer, 2002) either within the caldera or near the summit of Loihi Seamount from hydrothermal vent sites ranging in depth from 1150 to 1325 mbsl. Metagenomic DNA was extracted from these mat samples using the FastDNA Spin Kit following the manufacturer's protocol (Qbiogene, Irvine, CA). Extracted DNA was pooled, cleaned, and concentrated using Montáge PCR Centrifugal Filter Devices (Millipore, Bedford, MA). The DNA was then quantified using a NanoDrop spectrophotometer (Nanodrop Technologies, Wilmington, DE) and diluted to 10 ng·µl^−1^ using filter sterilized 10 mM Tris, pH 8.0. Three replicate SSU rDNA amplifications were performed, each using 50 ng of metagenomic DNA and bacterial Domain specific primers 68F and 1492R with PCR conditions as previously described [13]. The forward primer was labeled with 6-FAM (6-carboxyfluorescein) on the 5′ end. The amplicons were visually assayed for size by 1% agarose gel electrophoresis against a 1-kb ladder DNA size standard. Only reactions where corresponding negative controls yielded no amplification products were used. The remaining fluorescently-labeled PCR products were desalted and treated with eight tetrameric restriction enzymes as previously described in Chao et al. [51]. The fragments were separated by capillary electrophoresis using an ABI 3100 genetic analyzer using POP-6 with a 50 cm capillary array (Applied Biosystems, Foster City, CA). The fluorescently labeled 5′ terminal-restriction fragments were sized against the Genescan ROX-500 internal size standard using GeneMapper v3.7 (Applied Biosystems). Only fragments between 50 and 500 nucleotides were included in the analysis as this size range has been show to have the highest degree of precision [52]. Resulting electropherograms were then imported into BioNumerics v4.61 (Applied Maths, Austin, TX). Community fingerprints were compared in BioNumerics using the Pearson product-moment correlation [53]and unweighted pair group method with arithmetic mean (UPGMA) cluster analysis. The cophenetic correlation coefficient was calculated to assess the robustness of the assigned clusters.

## Supporting Information

Table S1 (0.05 MB DOC)

Text S1 (0.02 MB DOC)

Figure S1 A time series of images showing the effect of treating a stalk with oxalic acid. The oxalate reduces the iron oxides in the stalk causing shrinkage and substantial reduction in size of the stalk; however a remnant of material remains. In this stalk there was no visual change after 10 minutes. No cell was present on this stalk. For this experiment, stalks from a fresh culture of PV-1 were viewed at 1,000× by phase contrast microscopy and a drop of 0.3 M oxalic acid was placed at the edge of the coverslip allowing the oxalate to diffuse under the coverslip and reduce the Fe-oxides. Photomicrographs were captured at the indicated times. The bar = 5 μm.
(0.88 MB TIF)

Figure S2 Rep-PCR comparison of strains PV-1 and JV-1 with freshwater FeOB strains ES-2 (*‘Gallionella capsiferriformans’*), ES-1 (*‘Sideroxydans lithotrophicus’*), and BrT (*‘Sideroxydans paludicola’*). *Stenotrophomonas maltophilia* is included as a control. In duplicated runs PV-1 and JV-1 shared >98% similarity in their rep-PCR profiles. Rep-PCR profiles with % similarities <95% usually indicate unrelated strains.
(1.08 MB TIF)
