# Supplementary material for: A Novel Lineage of Proteobacteria Involved in Formation of Marine Fe-Oxidizing Microbial Mat Communities
Source: PLoS One. 2007 Aug 1;2(8):e667. doi: 10.1371/journal.pone.0000667 (PMC1930151; doi:10.1371/journal.pone.0000667)
Supplement: Table S1 — (0.05 MB DOC) [file pone.0000667.s001.doc]

**Supplementary Table 1. List of strains used for obtaining GyrB and RecA sequences for phylogenetic comparison to Mariprofundus.**

| **Species (and Strain or Deposit)** | **Accession** | **Class** |
| --- | --- | --- |
| **GyrB** |  |  |
| **Shigella flexneri 2a str. 2457T** | **NP_839178** | **Gammaproteobacteria** |
| **Escherichia coli K12** | **YP_026241** | **Gammaproteobacteria** |
| **Salmonella enterica subsp. enterica serovar Typhi str. CT18** | **NP_458107** | **Gammaproteobacteria** |
| **Erwinia carotovora subsp. atroseptica SCRI1043** | **YP_052523** | **Gammaproteobacteria** |
| **Yersinia pestis KIM** | **NP_671401** | **Gammaproteobacteria** |
| **Photorhabdus luminescens subsp. laumondii TTO1** | **NP_927380** | **Gammaproteobacteria** |
| **Buchnera aphidicola str. APS (Acyrthosiphon pisum)** | **NP_239852** | **Gammaproteobacteria** |
| **Candidatus Blochmannia floridanus** | **NP_878332** | **Gammaproteobacteria** |
| **Baumannia cicadellinicola str. Hc (Homalodisca coagulata)** | **YP_588600** | **Gammaproteobacteria** |
| **Pasteurella multocida subsp. multocida str. Pm70** | **NP_246415** | **Gammaproteobacteria** |
| **Haemophilus influenzae 86-028NP** | **YP_248279** | **Gammaproteobacteria** |
| **Mannheimia succiniciproducens MBEL55E** | **YP_089441** | **Gammaproteobacteria** |
| **Aeromonas hydrophila subsp. hydrophila ATCC 7966** | **YP_854532** | **Gammaproteobacteria** |
| **Idiomarina loihiensis L2TR** | **YP_154397** | **Gammaproteobacteria** |
| **Colwellia psychrerythraea 34H** | **YP_266786** | **Gammaproteobacteria** |
| **Shewanella oneidensis MR-1** | **NP_715653** | **Gammaproteobacteria** |
| **Pseudoalteromonas haloplanktis TAC125** | **YP_338561** | **Gammaproteobacteria** |
| **Acinetobacter sp. ADP1** | **YP_044811** | **Gammaproteobacteria** |
| **Psychrobacter arcticus 273-4** | **YP_263312** | **Gammaproteobacteria** |
| **Alcanivorax borkumensis SK2** | **YP_691724** | **Gammaproteobacteria** |
| **Pseudomonas aeruginosa PAO1** | **NP_064724** | **Gammaproteobacteria** |
| **Hahella chejuensis KCTC 2396** | **YP_431356** | **Gammaproteobacteria** |
| **Bordetella pertussis Tohama I** | **NP_879342** | **Betaproteobacteria** |
| **Burkholderia mallei ATCC 23344** | **YP_101864** | **Betaproteobacteria** |
| **Ralstonia eutropha H16** | **YP_724523** | **Betaproteobacteria** |
| **Azoarcus sp. EbN1** | **YP_158625** | **Betaproteobacteria** |
| **Nitrosomonas europaea ATCC 19718** | **NP_840104** | **Betaproteobacteria** |
| **Neisseria meningitidis MC58** | **NP_273269** | **Betaproteobacteria** |
| **Chromobacterium violaceum ATCC 12472** | **NP_899673** | **Betaproteobacteria** |
| **Agrobacterium tumefaciens str. C58** | **NP_530727** | **Alphaproteobacteria** |
| **Rhizobium leguminosarum bv. viciae 3841** | **YP_765616** | **Alphaproteobacteria** |
| **Sinorhizobium meliloti 1021** | **NP_384118** | **Alphaproteobacteria** |
| **Bartonella henselae str. Houston-1** | **YP_032908** | **Alphaproteobacteria** |
| **Brucella abortus biovar 1 str. 9-941** | **YP_220899** | **Alphaproteobacteria** |
| **Bradyrhizobium japonicum USDA 110** | **NP_767463** | **Alphaproteobacteria** |
| **Rhodopseudomonas palustris CGA009** | **NP_945360** | **Alphaproteobacteria** |
| **Silicibacter pomeroyi DSS-3** | **YP_165427** | **Alphaproteobacteria** |
| **Roseobacter denitrificans OCh 114** | **YP_680627** | **Alphaproteobacteria** |
| **Hyphomonas neptunium ATCC 15444** | **YP_759281** | **Alphaproteobacteria** |
| **Zymomonas mobilis subsp. mobilis ZM4** | **YP_163318** | **Alphaproteobacteria** |
| **Anaplasma phagocytophilum HZ** | **YP_505297** | **Alphaproteobacteria** |
| **Ehrlichia chaffeensis str. Arkansas** | **YP_507430** | **Alphaproteobacteria** |
| **Wolbachia endosymbiont of Drosophila melanogaster** | **NP_965933** | **Alphaproteobacteria** |
| **Neorickettsia sennetsu str. Miyayama** | **YP_506597** | **Alphaproteobacteria** |
| **Rickettsia typhi str. Wilmington** | **YP_067518** | **Alphaproteobacteria** |
| **Mariprofundis ferrooxydans** |  |  |
| **Myxococcus xanthus DK 1622** | **YP_628545** | **Deltaproteobacteria** |
| **Stigmatella aurantiaca DW4/3-1** | **ZP_01461072** | **Deltaproteobacteria** |
| **Desulfovibrio vulgaris subsp. vulgaris str. Hildenborough** | **YP_009228** | **Deltaproteobacteria** |
| **Geobacter sulfurreducens PCA** | **NP_951065** | **Deltaproteobacteria** |
| **Pelobacter carbinolicus DSM 2380** | **YP_355437** | **Deltaproteobacteria** |
| **Helicobacter pylori J99** | **NP_223171** | **Epsilonproteobacteria** |
| **Wolinella succinogenes DSM 1740** | **NP_906276** | **Epsilonproteobacteria** |
| **Campylobacter jejuni RM1221** | **YP_178032** | **Epsilonproteobacteria** |
| **RecA** |  |  |
| **Escherichia coli K12** | **NP_417179** | **Gammaproteobacteria** |
| **Salmonella enterica subsp. enterica serovar Typhi Ty2** | **NP_806431** | **Gammaproteobacteria** |
| **Photorhabdus luminescens subsp. laumondii TTO1** | **NP_928560** | **Gammaproteobacteria** |
| **Yersinia pestis CO92** | **NP_406773** | **Gammaproteobacteria** |
| **Erwinia carotovora subsp. atroseptica SCRI1043** | **YP_051458** | **Gammaproteobacteria** |
| **Wigglesworthia glossinidia endosymbiont of Glossina brevipal** | **NP_871238** | **Gammaproteobacteria** |
| **Shewanella oneidensis MR-1** | **NP_718983** | **Gammaproteobacteria** |
| **Vibrio vulnificus YJ016** | **NP_935598** | **Gammaproteobacteria** |
| **Photobacterium profundum SS9** | **YP_131186** | **Gammaproteobacteria** |
| **Colwellia psychrerythraea 34H** | **YP_270789** | **Gammaproteobacteria** |
| **Pasteurella multocida subsp. multocida str. Pm70** | **NP_246756** | **Gammaproteobacteria** |
| **Haemophilus influenzae Rd KW20** | **NP_438757** | **Gammaproteobacteria** |
| **Idiomarina loihiensis L2TR** | **YP_155132** | **Gammaproteobacteria** |
| **Pseudoalteromonas haloplanktis TAC125** | **YP_339221** | **Gammaproteobacteria** |
| **Pseudomonas syringae pv. tomato str. DC3000** | **NP_793795** | **Gammaproteobacteria** |
| **Hahella chejuensis KCTC 2396** | **YP_436332** | **Gammaproteobacteria** |
| **Nitrosomonas europaea ATCC 19718** | **NP_841954** | **Betaproteobacteria** |
| **Thiobacillus denitrificans ATCC 25259** | **YP_315955** | **Betaproteobacteria** |
| **Dechloromonas aromatica RCB** | **YP_287348** | **Betaproteobacteria** |
| **Burkholderia mallei ATCC 23344** | **YP_102098** | **Betaproteobacteria** |
| **Ralstonia eutropha JMP134** | **YP_294753** | **Betaproteobacteria** |
| **Bordetella pertussis Tohama I** | **NP_881173** | **Betaproteobacteria** |
| **Neisseria meningitidis Z2491** | **NP_284374** | **Betaproteobacteria** |
| **Chromobacterium violaceum ATCC 12472** | **NP_901277** | **Betaproteobacteria** |
| **Xanthomonas axonopodis pv. citri str. 306** | **NP_642071** | **Gammaproteobacteria** |
| **Xylella fastidiosa Temecula1** | **NP_778344** | **Gammaproteobacteria** |
| **Psychrobacter arcticus 273-4** | **YP_265014** | **Gammaproteobacteria** |
| **Coxiella burnetii RSA 493** | **NP_820055** | **Gammaproteobacteria** |
| **Legionella pneumophila subsp. pneumophila str. Philadelphia** | **YP_095827** | **Gammaproteobacteria** |
| **Methylococcus capsulatus str. Bath** | **YP_112918** | **Gammaproteobacteria** |
| **Mariprofundis ferrooxydans** |  |  |
| **Agrobacterium tumefaciens str. C58** | **NP_532554** | **Alphaproteobacteria** |
| **Rhizobium etli CFN 42** | **YP_469830** | **Alphaproteobacteria** |
| **Sinorhizobium meliloti 1021** | **NP_385905** | **Alphaproteobacteria** |
| **Mesorhizobium loti MAFF303099** | **NP_101919** | **Alphaproteobacteria** |
| **Brucella melitensis 16M** | **NP_539704** | **Alphaproteobacteria** |
| **Bartonella quintana str. Toulouse** | **YP_032418** | **Alphaproteobacteria** |
| **Rhodopseudomonas palustris CGA009** | **NP_949188** | **Alphaproteobacteria** |
| **Nitrobacter winogradskyi Nb-255** | **YP_317893** | **Alphaproteobacteria** |
| **Bradyrhizobium japonicum USDA 110** | **NP_772395** | **Alphaproteobacteria** |
| **Caulobacter crescentus CB15** | **NP_419903** | **Alphaproteobacteria** |
| **Zymomonas mobilis subsp. mobilis ZM4** | **YP_162901** | **Alphaproteobacteria** |
| **Oceanicaulis alexandrii HTCC2633** | **ZP_00952310** | **Alphaproteobacteria** |
| **Parvularcula bermudensis HTCC2503** | **ZP_01018219** | **Alphaproteobacteria** |
| **Silicibacter pomeroyi DSS-3** | **YP_167265** | **Alphaproteobacteria** |
| **Oceanicola batsensis HTCC2597** | **ZP_00999038** | **Alphaproteobacteria** |
| **Roseovarius nubinhibens ISM** | **ZP_00960604** | **Alphaproteobacteria** |
| **Rhodobacterales bacterium HTCC2654** | **ZP_01014749** | **Alphaproteobacteria** |
| **Loktanella vestfoldensis SKA53** | **ZP_01004742** | **Alphaproteobacteria** |
| **Rhodobacter sphaeroides 2.4.1** | **YP_353525** | **Alphaproteobacteria** |
| **Anaplasma marginale str. St. Maries** | **YP_153498** | **Alphaproteobacteria** |
| **Ehrlichia chaffeensis str. Arkansas** | **YP_507893** | **Alphaproteobacteria** |
| **Wolbachia endosymbiont of Drosophila melanogaster** | **NP_966773** | **Alphaproteobacteria** |
| **Neorickettsia sennetsu str. Miyayama** | **YP_506414** | **Alphaproteobacteria** |
| **Rickettsia prowazekii str. Madrid E** | **NP_221113** | **Alphaproteobacteria** |
| **Bdellovibrio bacteriovorus HD100** | **NP_967497** | **Deltaproteobacteria** |
| **Geobacter metallireducens GS-15** | **YP_383169** | **Deltaproteobacteria** |
| **Myxococcus xanthus DK 1622** | **YP_629698** | **Deltaproteobacteria** |
| **Helicobacter hepaticus ATCC 51449** | **NP_860164** | **Epsilonproteobacteria** |
| **Wolinella succinogenes DSM 1740** | **NP_907645** | **Epsilonproteobacteria** |
| **Campylobacter jejuni subsp. jejuni NCTC 11168** | **NP_282800** | **Epsilonproteobacteria** |
