# Supplementary material for: A Novel Lineage of Proteobacteria Involved in Formation of Marine Fe-Oxidizing Microbial Mat Communities
Source: PLoS One. 2007 Aug 1;2(8):e667. doi: 10.1371/journal.pone.0000667 (PMC1930151; doi:10.1371/journal.pone.0000667)
Supplement: Text S1 — (0.02 MB DOC) [file pone.0000667.s002.doc]

Supplemental Material.

Class, Order, and Family descriptions of *Mariprofundus ferroxydans* PV-1^T^

**Description of *Zetaproteobacteria* class nov.**

Zetaproteobacteria (ze.ta-Pro.te.o.bact’er.i.a) L. n. *zeta* sixth letter of the alphabet; N.L. *zeta-Proteobacteria* a sixth class of proteobacterium). This class designation is based on its deeply rooted phylogenetic position within the *Proteobacteria*. The class consists of one isolate *Mariprofundus ferrooxydans* and several environmental clones.

**Description of *Mariprofundales* ord. nov.**

*Mariprofundales* (Mar.i.pro.fund’a.les). L. masc. n. *Mariprofundus* the type genus of the order; N.L. -*ales* ending denoting an order; N.L. masc. n. *Mariprofundales* the order of *Mariprofundus*). The description is that of the class and in addition is characterized by organisms coming from the deep ocean.

**Description of *Mariprofundaceae* fam. nov.**

*Mariprofundaceae* (Mar.i.pro.fund’a.ce.ae). L. masc. n. *Mariprofundus* the type genus of the family; N.L. -*aceae* ending denoting a family; N.L. masc. n. *Mariprofundaceae* the family of *Mariprofundus.* The description is that of the order.
