# Supplementary figures and images for: A Novel Lineage of Proteobacteria Involved in Formation of Marine Fe-Oxidizing Microbial Mat Communities
Source: PLoS One. 2007 Aug 1;2(8):e667. doi: 10.1371/journal.pone.0000667 (PMC1930151; doi:10.1371/journal.pone.0000667)

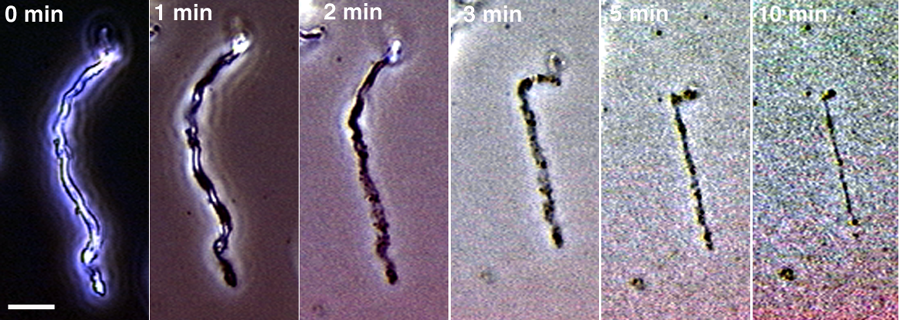

Supplement: Figure S1 — A time series of images showing the effect of treating a stalk with oxalic acid. The oxalate reduces the iron oxides in the stalk causing shrinkage and substantial reduction in size of the stalk; however a remnant of material remains. In this stalk there was no visual change after 10 minutes. No cell was present on this stalk. For this experiment, stalks from a fresh culture of PV-1 were viewed at 1,000× by phase contrast microscopy and a drop of 0.3 M oxalic acid was placed at the edge of the coverslip allowing the oxalate to diffuse under the coverslip and reduce the Fe-oxides. Photomicrographs were captured at the indicated times. The bar = 5 μm. (0.88 MB TIF) [file pone.0000667.s003.tif]

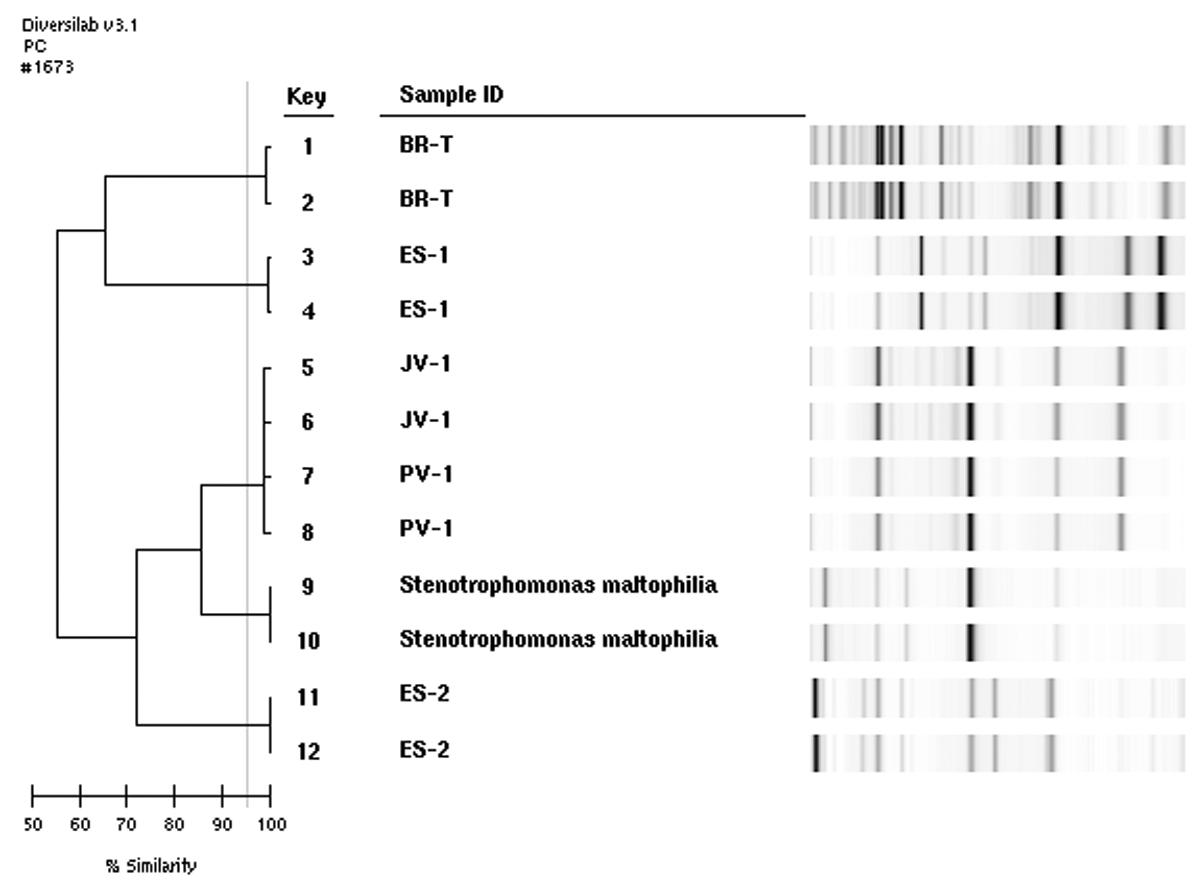

Supplement: Figure S2 — Rep-PCR comparison of strains PV-1 and JV-1 with freshwater FeOB strains ES-2 (‘Gallionella capsiferriformans’), ES-1 (‘Sideroxydans lithotrophicus’), and BrT (‘Sideroxydans paludicola’). Stenotrophomonas maltophilia is included as a control. In duplicated runs PV-1 and JV-1 shared >98% similarity in their rep-PCR profiles. Rep-PCR profiles with % similarities <95% usually indicate unrelated strains. (1.08 MB TIF) [file pone.0000667.s004.tif]
